# Automated Supra- and Infratentorial Brain Infarct Volume Estimation on Diffusion Weighted Imaging Using the RAPID Software

**DOI:** 10.3389/fneur.2022.907151

**Published:** 2022-07-08

**Authors:** Lehel Lakatos, Manuel Bolognese, Martin Müller, Mareike Österreich, Alexander von Hessling

**Affiliations:** ^1^Department of Neurology and Neurorehabilitation, Lucerne Kantonsspital, Lucerne, Switzerland; ^2^Department of Radiology (Section Neuroradiology), Lucerne Kantonsspital, Lucerne, Switzerland

**Keywords:** stroke, MRI, vertebrobasilar artery system, interobserver agreement, volume measure

## Abstract

**Purpose:**

The present computerized techniques have limits to estimate the ischemic lesion volume especially in vertebrobasilar ischemia (VBI) automatically. We investigated the ability of the RAPID AI (RAPID) software on diffusion-weighted imaging (DWI) to estimate the infarct size in VBI in comparison to supratentorial ischemia (STI).

**Methods:**

Among 123 stroke patients (39 women, 84 men, mean age 66 ± 11 years) having undergone DWI, 41 had had a VBI and 82 a STI. The infarct volume calculation by RAPID was compared to volume calculations by 2 neurologists using the ABC/2 method. For inter-reader and between-method analysis intraclass correlation coefficient (ICC), area under the curve (AUC) estimations, and Bland–Altman plots were used.

**Results:**

ICC between the two neurologists and each neurologist and RAPID were >0.946 (largest 95% *CI* boundaries 0.917–0.988) in the STI group, and > 0.757 (95% *CI* boundaries between 0.544 and 0.982) in the VBI group. In the STI group, AUC values ranged between 0.982 and 0.999 (95% *CI* 0.971–1) between the 2 neurologists and between 0.875 and 1 (95% *CI* 0.787–1) between the neurologists and RAPID; in the VBI group, they ranged between 0.925 and 0.965 (95% *CI* 0.801–1) between the neurologists, and between 0.788 and 0.931 (95% *CI* 0.663–1) between RAPID and the neurologists. Compared to the visual DWI interpretation by the neurologists, RAPID did not recognize a substantial number of infarct volumes of ≤ 2 ml.

**Conclusion:**

The ability of the RAPID software to depict strokes in the vertebrobasilar artery system seems close to its ability in the supratentorial brain tissue. However, small lesion volumes ≤ 2 ml remain still undetected in both brain areas.

## Introduction

Ischemic stroke in the vertebrobasilar (VB) artery distribution is less frequent compared to supratentorial infarctions but can be as devastating as a large hemispheric stroke. Intravenous thrombolysis (IVT) and intra-arterial thrombectomy are nowadays well established successful therapeutic approaches in supratentorial ischemia (STI). In VB ischemia (VBI), IVT can be applied successfully within the time window of 4.5 h after symptoms onset (1, 2). Mechanical thrombectomy in the vertebral or basilar artery is performed as live-saving procedures but without a clear basis of superiority in randomized clinical trials against iv thrombolysis and best medical care (2, 3). One reason could be that the present imaging techniques [computerized tomography (CT), magnetic resonance imaging (MRI)] struggle to apply the core/penumbra mismatch concept to VBI for a better patient characterization. For example, Garcia-Esperon et al. (4) reported a sensitivity of about 25% of lacunar strokes in the VB system to be recognized by multi-modal perfusion CT. Cerebellar infarction may be larger, but a good ability of multi-model perfusion CT to detect cerebellar infarction was found only for infarcts > 4–5 ml (5, 6). Small structure size, the availability and the duration of the examination (MRI), the poor spatial resolution (CT), physiological variability of cerebral perfusion in this area (7), and the technical difficulties to measure cerebral blood flow correctly in this anatomical region hinder the application of the stroke mismatch concept to a broad range of VBS.

Several software packages exist (among others e.g., RAPID^®^, iSchemaView; Olea Sphere^®^, Olea Medical) to estimate infarct core and penumbra size in STI automatically (8). It is not known how these software packages perform in VBI on MRI technology. To explore this field, we performed as a first step an explorative study in stroke patients to compare the ability of the RAPID software package to estimate the stroke lesion volume on diffusion-weighted imaging (DWI) in VBI in comparison to two stroke-trained neurologists. By applying the same analysis to STI, we sought to generate a reference for reproducibility and reliability expectations. Given the good spatial resolution of present MRI technology to detect even small infarctions, our hypothesis was that the software package can estimate the infarct size in the VB system comparably to infarcts in the supratentorial brain tissue.

## Materials and Methods

The study was approved by the Ethics Committee of Northwest and Central Switzerland (PB_2016-01719) and was performed in accordance with the Declaration of Helsinki, using good standards of clinical practice. It is a part of the larger trial registered at ClinicalTrials.gov NCT04611672. Written informed consent was obtained from all participants. All data are available on request from the authors.

The Lucerne hospital is a large tertiary teaching hospital with a full stroke center service. All patients with a stroke syndrome receive standardized care with initially a focused clinical examination followed by a multimodal cranial computed tomography [CT; Siemens Force, Edge or XCeed CT; native, followed by perfusion CT [postprocessed by Syngo. *via* Rapid AI Software (RAPID)], and CT angiography]. If indicated, iv thrombolysis and/or arterial thrombectomy follows immediately. All patients with a stroke syndrome are transferred to the stroke unit for close clinical monitoring. Extensive ultrasound examinations of the extra- and transcranial arteries (including noninvasive auto-regulatory measurements) are performed, and an echocardiography and a brain MRI follow within 48 h after hospitalization. At our institution, only the RAPID software is in use and well functioning; the maintenance of other software packages (such as the Olea Sphere^®^, Olea Medical) was not considered due to the overall high costs.

### MRI Investigation

For MRI examinations, three MR-Scanners are present; a Siemens Vida fit (3 Tesla), a Siemens Aera (1, 5 Tesla), and a Philips Achieva (3 Tesla). For the automated stroke volume estimations, we used the RAPID software. The patients were randomly assigned to the scanners. While the raw data of the Siemens MRI were provided to allow for RAPID analysis, the Philips MRI was not able to provide its raw data accordingly (The DWI b1000 images are not correctly scaled in relation to the b0 images and, therefore, do not allow calculation of ADC maps by the RAPID software). Thus, patients examined with the Philips MRI had to be excluded from the analysis. Consequently, for this retrospective analysis, we could recruit between January and September 2020, 123 consecutive patients with acute cerebral ischemic syndromes examined at the two mentioned Siemens MRI scanners.

We focused our analysis on DWI because DWI has the capability of highlighting acute ischemic stroke probably better than any other MRI sequences. The DWI images were acquired in the axial plane with b-values of 0, 500, and 1,000 s/mm^2^ as a single-shot EPI Sequence (TR/TE 7,600/78 ms), isotorpic diffusion weighting, a matrix of 192 × 192, and a FOV of 220 × 220 mm^2^ with a slice thickness of 4 mm. For each b-value, three gradient directions were used. b0 and b1000 images were transferred to the Rapid AI Server and postprocessing was done with standard settings (Software version 4.9.1.1). On the Rapid Server, the ADC maps were calculated based on the following data acquisition: with the 3T Vida scanner, only b0 and b1000 images were acquired, with the 1.5T Aera scanner b500 images were additionally acquired. The detection and 3D volumetry of the infarcts were done with standard parameters of RAPID assigning ADC values of below 620 × 10^−6^ mm^2^/s to infarcted tissue. Areas of ADC values below 620 × 10^−6^ mm^2^/s were transferred onto the maps and highlighted in color. Volumetry of infarct was also done automated by the RAPID software, and data of infarcted brain tissue (if detected) were noted in an excel database.

**Figure 1:**
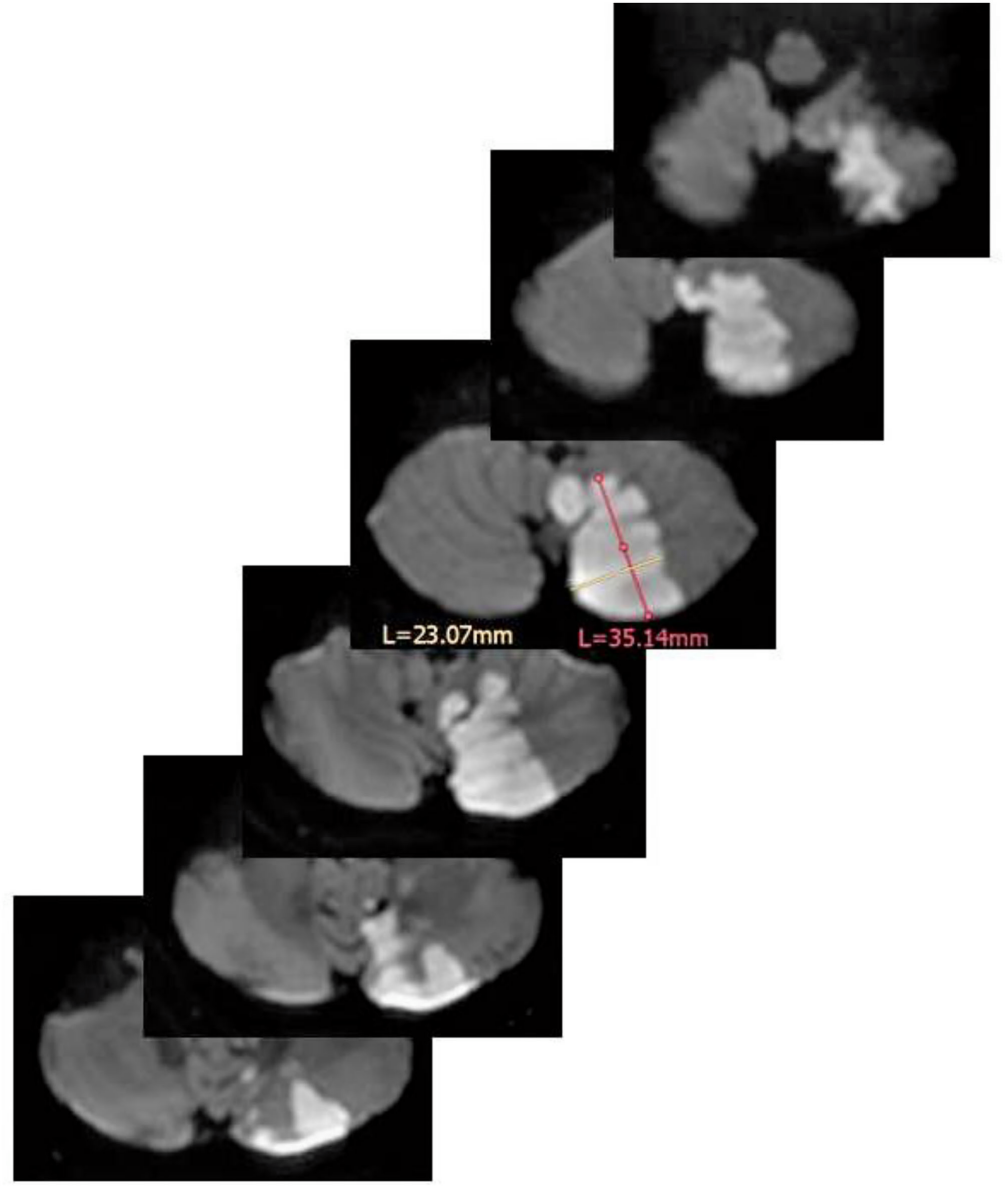
Volume estimation of a left PICA infarction by means of the ABC/2 method. In the scan with the largest infarction A (in magenta) = 3.51 cm, B (in yellow) = 2.30 cm. Of note, A and B should stand perpendicular to each other. The thickness of each scan is 0.4 cm. The resulting volume is: (3.51 × 2.30 × 0.4 cm × 6)/2 = 9.68 cm^3^ (=9.68 ml). L = length of measurements in mm for A and B.

On the DWI scans, the two neurologists calculated independently from one another the DWI lesion volume by the ABC/2 method which provides good reliability and reproducibility (9, 10). In this method, the infarcts size is calculated as follows: the area (A × B) of the infarct size is measured at the plane with the largest infarct expansion; this area is then multiplied by the number of scan slices on which the infarct is visible multiplied by the slice thickness (C); the resulting volume is then divided by 2. An example is provided in Figure 1. When no lesion was detected, the MRI was classified 0 ml volume. If several infarct lesions were present, the volumes of all lesions were added to one infarct volume.

The study population (Table 1) consists of 39 women, 84 men with a mean age of 66 ± 11 years.

**Table 1:** Study population characteristics.

	**Patients with VBI (*n* = 41)**	**Patients with STI (*n* = 82)**
Age (years, mean ± SD)	65 ± 16	66 ± 16
Female/male	9/32	30/52
Transient ischaemic attack	5	12
Stroke	36	70
High Blood Pressure	22	46
Diabetes mellitus	14*	13
Smoking	12	19
Dyslipidaemia	30	63
BMI		
<25 (normal) 25-29.9 (pre-obesity) >30 (obesity)	19 13 9	32 40 10
Pathogenesis according to TOAST (11)		
Cardioembolic Large artery disease Small vessel disease Unknown	5 19* 12 5	18 14 16 34*

VBI, vertebrobasilar artery ischaemia; STI, supratentorial ischaemia; SD, standard deviation; Fisher's exact test ^*^p < 0.01.

For definition, we considered a transient ischemic event, a TIA only if DWI did not show a lesion corresponding to the clinical deficit which completely resolved within 24 h. If a lesion was present, we considered any clinical transient event a stroke (with good clinical recovery). Before any measurements were performed both neurologists reviewed independently from one another, the DWI imaging of each patient as to whether there was a lesion present or not. Apart from one MRI, they agreed in 122 patients on the presence or absence of a fresh lesion. The one MRI with dissens was re-evaluated by both, and the final verdict of both in consents was that a lesion was present.

Forty-one of the 123 patients had had a VBI [5 transient ischemic attacks (TIA), 36 strokes (cerebellar 26, medulla oblongata 4, midbrain 3, and pons 3], 82 a STI (12 TIA, 70 strokes). The population's baseline characteristics are provided in Table 1. Compared to the STI group, the patients in the VBI group were less likely to suffer from ischemic events of unknown origin (OD 0.196, 95% Confidence interval [*CI*] 0.06–0.55), but more likely to suffer from diabetes mellitus (*OD* 2.75; 95% *CI* 1.14–6.61) or large artery disease in the vertebrobasilar artery system [>50% stenosis or occlusions; *OD* 4.19, 95% *CI* 1.80–9.72]).

### Statistical Analysis

For statistical analysis (Matlab Statistic toolbox, Mathworks, Natick, Massachusetts, USA), we used the Kolmogorov–Smirnov Test to test the variables for normal distribution. All volumetric data were not normally distributed. Fisher's exact test and odds ratio calculations were used to compare the frequency of baseline characteristics in the VBS and STS groups. The non-parametric Wilcoxon Signed-rank test was used to compare the means of the groups containing RAPID measurements; we report them as median and interquartile ranges. To examine reliability and reproducibility, we used Bland–Altman plots (12), intraclass correlation coefficient (ICC) analysis, and the areas under the curve (AUC) of receiver-operated curves which are based on sensitivity and 1-specificity characteristics.

The ICC values (ranging from 0 to 1) were interpreted as follows: excellent agreement ICC ≥ 0.90, good agreement ICC ≥ 0.75, moderate agreement 0.75 > ICC ≥ 0.50, and poor agreement ICC < 0.5 (13). The AUC values were interpreted as AUC 0.9–1 very good, 0.8–0.9 good, 0.7–0.8 fair, 0.6–0.7 poor, <0.6 fail (14).

## Results

### Supratentorial Ischemia Group

For an overall estimating of the stroke volumes, the intraclass correlation coefficient between neurologists 1 and 2 was 0.981 (95% *CI*, 0.970–0.988, *F* = 51.7, df 81); it was 0.946 (95% *CI* 0.917–0.965, *F* = 18.4, df 81) between neurologist 1 and the RAPID software, and 0.947 (95% *CI*, 0.917–0.966, *F* = 18.5, df 81) between neurologist 2 and RAPID. The AUC results between the two neurologists and the RAPID software are provided in Table 2 with neurologist 1 as the reference measurement, and Table 3 with the RAPID software results as the reference. As a grand average, the AUC values indicate excellent reader agreement between all three measures.

**Table 2:** Areas under the curve (AUC) of receiver operated curves at different stroke volumes for supratentorial stroke volumes with neurologist1 as reference.

	**Neurol2**	**RAPID**
	**AUC (95% CI)**	**AUC (95% CI)**
Cutoff value in mL measured by Neurol1	
≤ 1 vs. >1	0.982 (0.961, 1)	0.875 (0.787, 0.963)
≤ 3 vs. >3	0.989 (0.968, 1)	0.947 (0.881, 1)
≤ 5 vs. >5	0.999 (0.995, 1)	0.958 (0.896, 1)
≤ 7 vs. >7	0.999 (0.995, 1)	0.965 (0.913, 1)
≤ 9 vs. >9	0.995 (0.985, 1)	0.953 (0.894, 1)
≤ 11 vs. > 11	0.999 (0.997, 1)	0.976 (0.947, 1)
≤ 20 vs. > 20	0.999 (0.996, 1)	0.968 (0.934, 1)
≤ 50 vs. >50	0.990 (0.970, 1)	1 (1, 1)
≤ 70 vs. >70	0.990 (0.970, 1)	1 (1, 1)

*Neurol1 and Neurol2, Neurologist 1 and 2; RAPID, RAPID software*.

**Table 3:** The AUC of receiver operated curves at different stroke volumes for supratentorial stroke volumes with RAPID as reference.

	**Neurol1**	**Neurol2**
	**AUC (95% CI)**	**AUC (95% CI)**
Cutoff value in mL measured by RAPID
≤ 1 vs >1	0.989 (0.975, 1)	0.993 (0.981, 1)
≤ 3 vs. >3	0.989 (0.975, 1)	0.993 (0.981, 1)
≤ 5 vs. >5	0.992 (0.982, 1)	0.993 (0.982, 1)
≤ 7 vs. >7	0.995 (0.986, 1)	0.996 (0.988, 1)
≤ 9 vs. >9	0.983 (0.957, 1)	0.981 (0.950, 1)
≤ 11 vs. > 11	0.983 (0.957, 1)	0.981 (0.950, 1)
≤ 20 vs. > 20	0.976 (0.948, 1)	0.969 (0.935, 1)
≤ 50 vs. >50	1 (1,1)	0.990 (0.970, 1)
≤ 70 vs. >70	1 (1,1)	1 (1, 1)

*Neurol1 and Neurol2, Neurologist 1 and 2; RAPID, RAPID software*.

**Figure 2:**
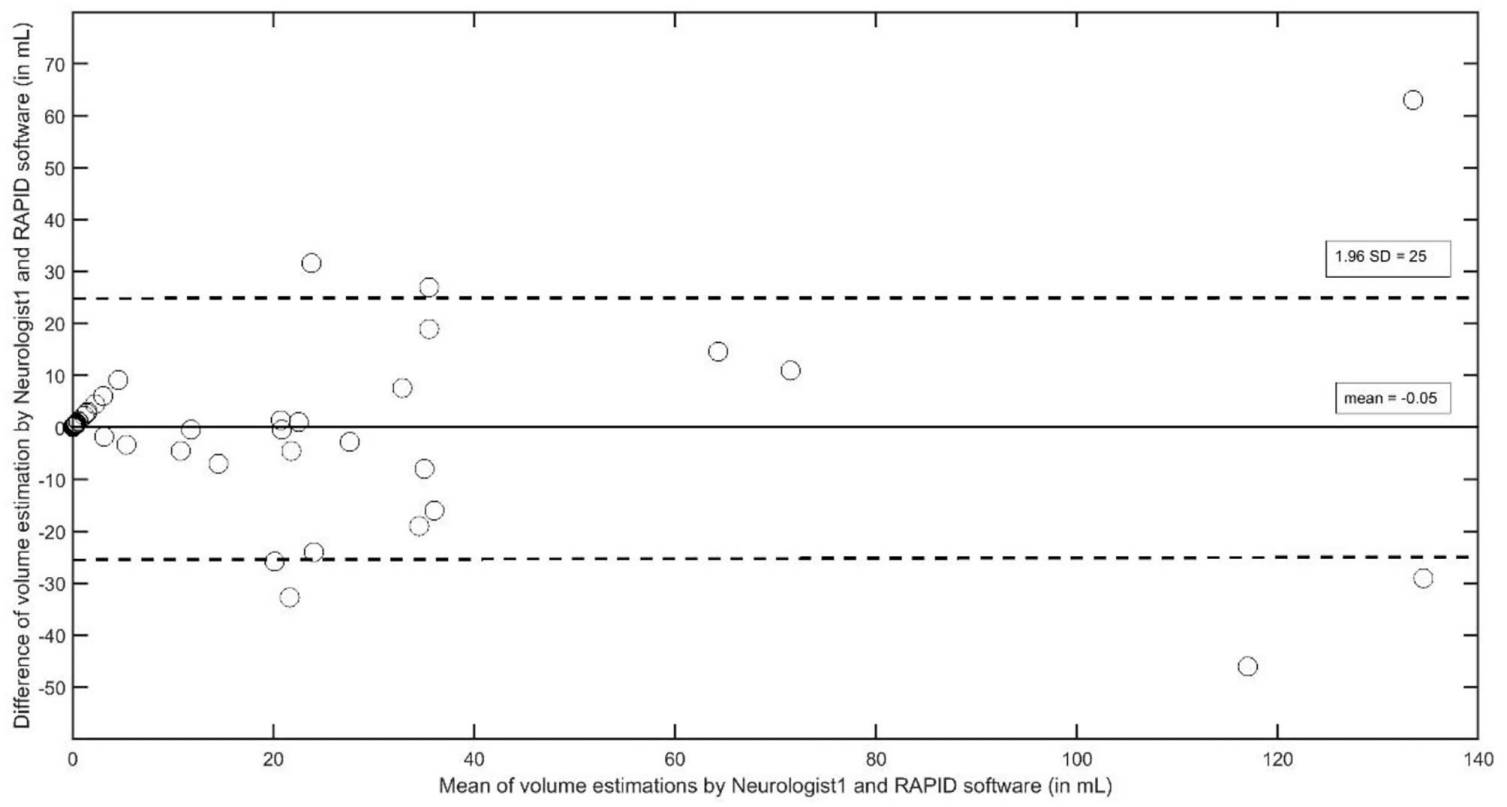
The Bland–Altman plot to analyze interobserver agreement between Neurologist1 and the RAPID software to estimate stroke volumes in supratentorial strokes.

The Bland–Altman plot between volume estimations by Neurologist 1 and the RAPID software (Figure 2) indicates that outliners beyond the 1.96 SD limits are rare but may occur more frequently at larger stroke volumes; additionally, it seems that the size of the infract volumes seems to spread wider with increasing infarct size (>20 ml). This result may contribute to earlier observations that the ability of automated software to estimate the infarct volume may depend on the infarct's anatomical location and its size (15). For such a detailed analysis, we arbitrarily classified the lesions into three groups: the first group (*n* = 26) consists of patients with cortical infarcts plus basal ganglia infarcts with a total infarct volume of more than 2 ml (defined by ABC/2 of neurologist 1); group two (*n* = 26) contains infarcts of cortical plus basal ganglia areas which, however, totaled to a total infarct volume of <2 ml (small infarct group); the third group (*n* = 18) contains subcortical white matter infarcts. Pathophysiologically, we assume that cortical and basal ganglia infarcts are more likely to be of embolic origin, while subcortical infarcts are more likely to be of microangiopathic origin. In the group of cortical/basal ganglia infarcts, >2 ml infarct volumes were significantly larger with the ABC/2 method (median 27; interquartile range 11–47) compared to the RAPID results (median 12.5, interquartile range 0–28; *p* < 0.01, *z* = −2.83, df 26). In the group of small cortical/basal ganglia infarcts and in the group of white matter infarcts, both methods did not differ [cortical/basal ganglia infarct < 2 ml: ABC/2 median 0.7 (interquartile range 0.35–1.7); RAPID median 0 (interquartile range 0–2); white matter infarcts: ABC/2 median 0.32 (interquartile range 0.09–0.53); RAPID median 0 (interquartile range 0–5)].

It is, however, worthwhile to note that the RAPID software did not recognize 45 of the 70 strokes (Table 4); the not recognized strokes were overwhelmingly in the groups of white matter strokes and in the group of cortical/basal ganglia strokes < 2 ml.

**Table 4:** Number of supratentorial automated detected diffusion weighted imaging (DWI) lesions in comparison to the visual detected ones by the trained neurologists.

	**Neurologists**		**Total**

**RAPID**	**No**	**Yes**	
No	12	45	57
Yes	0	25	25
Total	12	70	82

*RAPID, RAPID software; No, infarct not recognized; Yes, infarct recognized; 12 patients had had a TIA which, by definition, did not presented with DWI lesions*.

### Vertebrobasilar Ischemia Group

**Figure 3:**
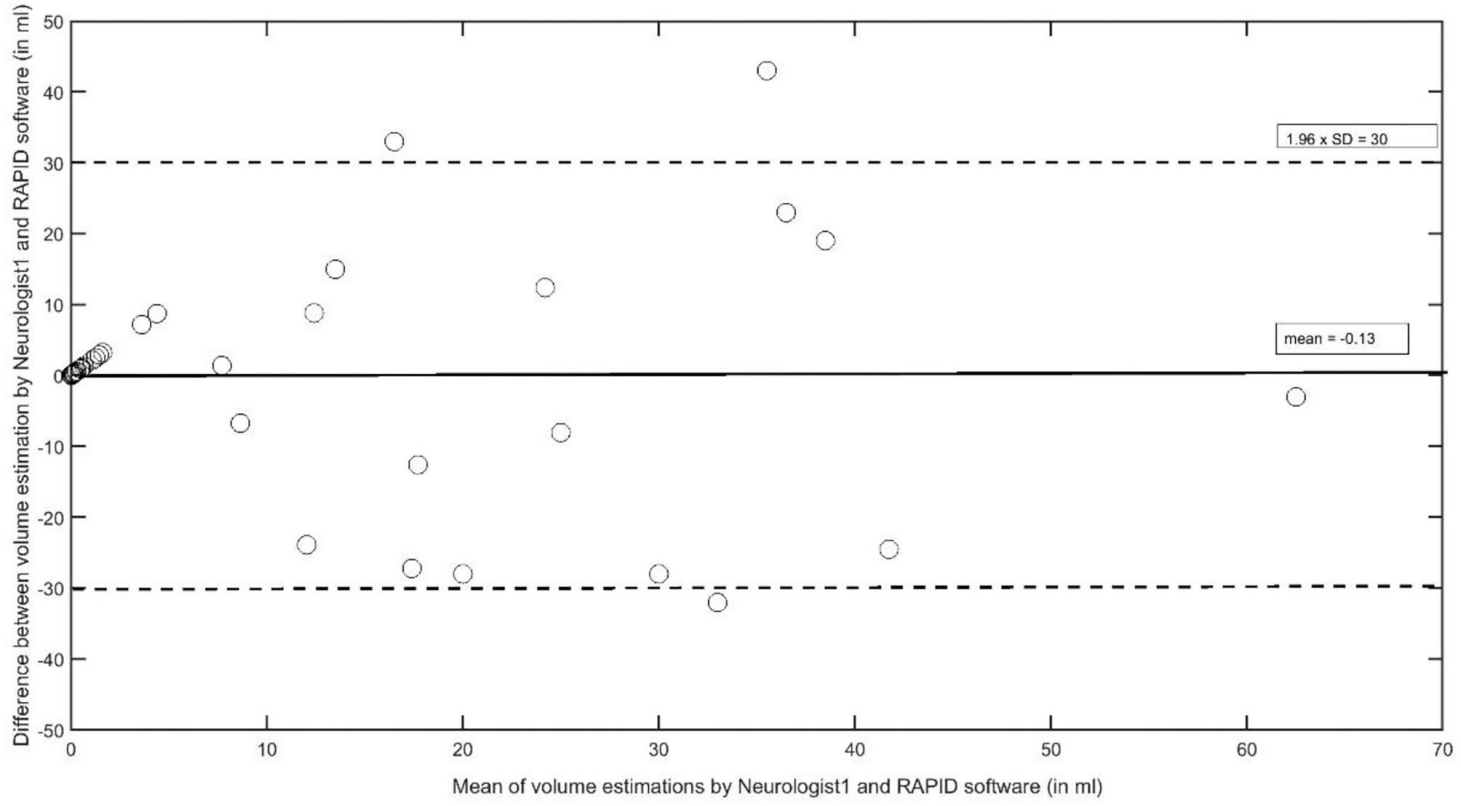
The Bland–Altman plot to analyze interobserver agreement between Neurologist1 and the RAPID software to estimate stroke volumes in the vertebrobasilar artery system.

For an estimation of the overall agreement regarding the VB stroke volume, the intraclass correlation coefficients between neurologists 1 and 2 was 0.967 (95% *CI*, 0.938–0.982, *F* = 30.1, df 40); it was 0.757 (95% *CI* 0.544–0.870, *F* = 4.1, df 40) between neurologist 1 and the RAPID software, and 0.762 (95% *CI*, 0.553–0.873, *F* = 4.1, df 40) between neurologist 2 and RAPID. The AUC results between the two neurologists and the RAPID software are provided in Table 5 with neurologist 1 as the reference measurement, and Table 6 with the RAPID software results as the reference. As a grand average, the AUC values indicate good to very good reader agreement between all three measures. The Bland–Altman plot (Figure 3) between volumes estimations by Neurologist 1 and the RAPID software shows again that outliners are rare; the infarct size-dependent spreading between the two observers seems not so present compared to STI.

**Table 5:** The AUC of receiver operated curves at different stroke volumes for vertebrobasilar stroke volumes with neurologist 1 as reference.

	**Neurol2**	**RAPID**
	**AUC (95% CI)**	**AUC (95% CI)**
Cutoff value in mL measured by Neurol1
≤ 1 vs. >1	0.969 (0.900, 1)	0.940 (0.869, 1)
≤ 3 vs. >3	0.901 (0.801, 1)	0.963 (0.908, 1)
≤ 5 vs. >5	0.935 (0.853, 1)	0.947 (0.878, 1)
≤ 7 vs. >7	0.940 (0.865, 1)	0.903 (0.817, 0.989)
≤ 9 vs. >9	0.925 (0.841, 1)	0.918 (0.841, 0.996)
≤ 11 vs. > 11	0.925 (0.841, 1)	0.918 (0.841, 0.996)
≤ 20 vs. > 20	0.965 (0.916, 1)	0.892 (0.810, 0.974)

*Neurol1 and Neurol2, Neurologist 1 and 2; RAPID, RAPID software*.

**Table 6:** The AUC of receiver operated curves at different stroke volumes for vertebrobasilar stroke volumes with RAPID as reference.

	**Neurol1**	**Neurol2**
	**AUC (95% CI)**	**AUC (95% CI)**
Cutoff value in mL measured by RAPID
≤ 1 vs. >1	0.809 (0.688, 0.930)	0.788 (0.663, 0.914)
≤ 3 vs. >3	0.913 (0.825, 1)	0.851 (0.740, 0.962)
≤ 5 vs. >5	0.931 (0.852, 1)	0.892 (0.795, 0.990)
≤ 7 vs. >7	0.890 (0.799, 0.981)	0.878 (0.780, 976)
≤ 9 vs. >9	0.931 (0.860, 1)	0.897 (0.809, 0.985)
≤ 11 vs. > 11	0.931 (0.860, 1)	0.897 (0.809, 0.985)
≤ 20 vs. > 20	0.931 (0.839, 0.986)	0.936 (0.872, 1)

*Neurol1 and Neurol2, Neurologist 1 and 2; RAPID, RAPID software*.

For a more detailed analysis with respect to infarct allocation, we grouped the strokes in 26 cerebellar and in 10 non-cerebellar strokes. Of them, RAPID detected 14 in the cerebellar group and only 1 in the non-cerebellar group (Table 7). In the cerebellar infarct group, ABC/2 volume by neurologist1 (median 16.8; interquartile range 5.6–31) did not differ from RAPID volume measurement (median 24; interquartile range 6.5–23). The ABC/2 infarct volume in the non-cerebellar group was 0.28 (interquartile range 0.08–0.55) by neurologist1.

**Table 7:** Number of infratentorial automated detected DWI lesions in comparison to the visual detected ones by the trained neurologists.

	**Neurologists**		**Total**

**RAPID**	**No**	**Yes**	
No	5	20	25
Yes	0	16	16
Total	5	36	41

*RAPID, RAPID software; No, infarct not recognized; Yes, infarct recognized; 5 patients had had a TIA which, by definition, did not presented with DWI lesions*.

## Discussion

Using ICC and AUC methods, our inter-reader and between-method agreements were mostly very good and correspond to reports in which large hemispheric strokes were compared (10, 15–18). The Bland-Altman plot showed that outliners beyond the ±1.96 SD limits were rare. Our analysis indicates that the ABC/2 method for stroke volume estimation can be similar effective in VBI.

A recognizable difference between STI and VBI lesion size estimation was found for the absolute size in ml. While there was no difference in the VBI group, the ABC/2 method overestimated the infarct sizes in the STI group with large infarctions roughly by twice. Closer results have been reported when the area of the infarct expansion is calculated at each scan separately and all areas are added up (10). It is, however, unclear whether this influences the decision-making toward thrombectomy because each kind of the applied ABC/2 method has its inherent-size calculation problems with respect to the final core/penumbra ratio. We rather believe that errors in volume estimations by the ABC/2 method in the way we performed it are the more likely in the supratentorial brain, the larger the mathematical error can be by multiplying one fixed area over many scans necessary to depict the whole anatomic infarct volume. In the posterior fossa, such a mathematical error seems more limited due to the anatomically restricted volume expansion possibility.

### Are Our Results Clinically Useful?

In most clinical routine settings (like ours) with its usually applied multimodal CT and/or MRI with DWI scanning, the initial ischemic damage in VBI is rarely quantified with the purpose to stratify any therapeutic intervention, especially thrombolysis. One score is the pc-ASPECT score applied on CT or MRI imaging (19, 20). This score is semiquantitative and scores only which vascular region is affected by ischemia but does not provide volume size estimations. Quantification with MRI seems more accurate when co-registration techniques are applied; however, such techniques are rarely available in the emergency situations. Nevertheless, they might be helpful to overcome the known tendency of the ABC/2 method to overestimate infarct size in STI. According to our results that might be different in VBI where we did not recognize an infarct size overestimation by the ABC/2 method. With the present mostly available techniques, the ABC/2 approach seems a rapid and an easy to apply approach to stratifications in VBI, if the initial imaging technique is MRI with DWI.

The VBI infarct size calculation was not different between the RAPID software and the two neurologists in the cerebellar stroke group indicating that the ABC/2 method seems as useful as the RAPID software to estimate the infarct core. We, therefore, consider a follow-up study that includes MRI perfusion to investigate whether the ABC/2 method is comparable to RAPID to estimate the tissue at risk. If confirmed, the ABC/2 method has the prospect to be used in cerebellar stroke to indicate recanalization therapy in a not live-saving situation (21). Regarding brainstem infarction, our results demonstrated that the RAPID software is at present inferior to the visual analysis of a trained neurologist/neuroradiologist to detect brainstem lesions. It seems, at least for now, that the use of visual MRI interpretation and the use of the ABC/2 method on MRIs in the vertebrobasilar artery region is likely to be more attractive for clinical trials than the use of automated software.

The major limits of our study seem twofold. First, in the intended use of the RAPID software. In STI, the severity of the neurological deficit increases with the amount of infarcted brain tissue. The RAPID software is designated to recognize large amounts of ischemic brain tissue with the aim to help to avoid such devastating strokes by thrombolysis/thrombectomy. In this context, the missing of smaller parts of the ischemic tissue is of less relevance. Whether this can also be applied to the much smaller infratentorial brain region, is unproven and doubtful. Additionally, it is not clear whether other software packages will have the same shortcomings. It is, therefore, evident to try to strengthen our findings by using other software packages. If other researchers like to repeat this study with another software package, we are willing to share our original data. Additionally, the use of neuronal networks by which the amount of detected lesions < 1 ml (14) is much larger, could be of help. Secondly, as mentioned above co-registration methods as such used in fMRI might calculate brain (infarct) volumes more accurately. However, such methods are at present not part of emergency settings that are clinically optimized to spare time. Such methods may, therefore, be a promising research direction but they have to consider time and they have to overcome technical difficulties in that (according to our experience) the DWI voxel data structure offers only a poor quality after uploading in a three-dimensional space to allow for calculation of good Dice coefficients.

## Data Availability Statement

The original contributions presented in the study are included in the article/supplementary material, further inquiries can be directed to the corresponding author/s.

## Ethics Statement

The studies involving human participants were reviewed and approved by Ethics Committee of Northwest and Central Switzerland (PB_2016-01719). The patients/participants provided their written informed consent to participate in this study.

## Author Contributions

LL: data analysis and writing. MB: data analysis and important intellectual content. MM: study concept, data analysis, and writing. MÖ: study concept. AH: study concept and RAPID analysis writing. All authors contributed to the article and approved the submitted version.

## Conflict of Interest

The authors declare that the research was conducted in the absence of any commercial or financial relationships that could be construed as a potential conflict of interest.

## Publisher's Note

All claims expressed in this article are solely those of the authors and do not necessarily represent those of their affiliated organizations, or those of the publisher, the editors and the reviewers. Any product that may be evaluated in this article, or claim that may be made by its manufacturer, is not guaranteed or endorsed by the publisher.
